# The Impact of *Enterococcus* spp. in the Immunocompromised Host: A Comprehensive Review

**DOI:** 10.3390/pathogens13050409

**Published:** 2024-05-15

**Authors:** Giuseppe Sangiorgio, Maddalena Calvo, Giuseppe Migliorisi, Floriana Campanile, Stefania Stefani

**Affiliations:** 1 Department of Biomedical and Biotechnological Sciences, University of Catania, Via Santa Sofia 97, 95123 Catania, Italy; f.campanile@unict.it (F.C.); stefania.stefani@unict.it (S.S.); 2 U.O.C. Laboratory Analysis Unit, University Hospital Policlinico-San Marco, Via Santa Sofia 78, 95123 Catania, Italy; maddalenacalvo@gmail.com (M.C.); gpp.miglio@gmail.com (G.M.)

**Keywords:** *Enterococcus*, immunocompromised host, infection, pathogenicity

## Abstract

The immunocompromised host is usually vulnerable to infectious diseases due to broad-spectrum treatments and immunological dysregulation. The *Enterococcus* genus consists of normal gut commensals, which acquire a leading role in infective processes among individuals with compromised immune systems. These microorganisms may express a potential virulence and resistance spectrum, enabling their function as severe pathogens. The *Enterococcus* spp. infections in immunocompromised hosts appear to be difficult to resolve due to the immunological response impairment and the possibility of facing antimicrobial-resistant strains. As regards the related risk factors, several data demonstrated that prior antibiotic exposure, medical device insertion, prolonged hospitalization and surgical interventions may lead to *Enterococcus* overgrowth, antibiotic resistance and spread among critical healthcare settings. Herein, we present a comprehensive review of *Enterococcus* spp. in the immunocompromised host, summarizing the available knowledge about virulence factors, antimicrobial-resistance mechanisms and host-pathogen interaction. The review ultimately yearns for more substantial support to further investigations about enterococcal infections and immunocompromised host response.

## 1. Introduction

Microorganisms belonging to the *Enterococcus* genus have long been recognized as important human gut commensals. Despite this assumption, their ability to persist in the environment and resist disinfection procedures has led to their widespread distribution in clinical settings [1]. Particularly, *Enterococcus faecalis* and *Enterococcus faecium* are notable for causing considerable management issues due to episodes of antimicrobial resistance. Their interactions with the host, including alterations in host cell signalling pathways and evasion of immune responses, further increase their pathogenicity and ability to cause long-lasting infections [2,3]. It is crucial to understand the characteristics, patterns of antibiotic resistance, and host interactions of *E. faecalis* and *E. faecium* for several reasons. Firstly, these bacteria are responsible for a wide range of infections, including urinary tract infections, bacteraemia, endocarditis, intra-abdominal infections, and surgical site infections. Furthermore, the clinical significance of *Enterococcus* species is heightened by their ability to cause both community-acquired and healthcare-associated illnesses [4,5,6]. Secondly, the emergence and spread of antibiotic-resistant strains, particularly those resistant to vancomycin, pose a significant threat to public health. This resistance profile complicates therapeutic approaches, increases the risk of treatment failure, and underscores the urgent need for effective antimicrobial stewardship procedures [7,8,9]. Thirdly, the development and persistence of infections are significantly influenced by the relationship between *Enterococcus* species and the host.

*Enterococcus* species is a leading cause of nosocomial infections, especially in immunocompromised patients. The rise in vancomycin-resistant *Enterococcus* (VRE) cases across Europe is particularly concerning, with resistance even emerging against last-resort antibiotics, severely limiting treatment options [10]. Infections caused by multidrug-resistant (MDR) enterococcal strains are associated with substantial economic burdens and higher rates of morbidity and mortality compared to those caused by susceptible strains [11,12]. Additionally, *enterococci* are highly resilient, withstanding harsh conditions, resisting biocides, forming biofilms, and possessing high genetic adaptability, all of which pose challenges to decontamination efforts. Consequently, enterococci serve as significant sources of nosocomial outbreaks and the dissemination of resistance genes. Diagnosis of antimicrobial tolerance in persistent infections in immunocompromised hosts, or in body sites with restricted immune access, is of particular concern as strong, effective bactericidal antimicrobials are necessary in these circumstances [13]. Moreover, enterococci play a significant role in maintaining intestinal homeostasis, contributing to continuous immune system stimulation [14]. Understanding these interactions between the host and these microorganisms can help identify new therapeutic targets and preventative measures for enterococcal infections [15,16].

In this review, we aim to provide a comprehensive insight into *Enterococcus* species as human pathogen and how they interact with immunocompromised patients, focusing on its pathogenicity, antibiotic resistance, and interaction mechanisms with the host.

## 2. Epidemiological Patterns of Enterococcal Infections

According to the World Health Organization (WHO) recent data, *Enterococcus* species mortality rate waves between 14.3% and 32.3% [17]. In 2020, vancomycin resistance in *E. faecium* showed significant variation across European countries and regions, with seven (18%) reporting percentages below 1%, including Finland, France, Iceland, the Netherlands, Norway, Sweden, and Ukraine. Conversely, 13 countries/areas (34%) reported antimicrobial resistance (AMR) percentages equal to or above 25%, with four (11% of 38 countries/areas) reporting percentages equal to or above 50%, including Bosnia and Herzegovina, Lithuania, North Macedonia, and Serbia [10]. Globally, the incidence of *E. faecalis* infections has remained relatively stable over the past two decades, although literary data report some localized outbreaks [18,19,20,21,22]. In Europe, according to the European Center for Disease Prevention and Control (ECDC) Surveillance Atlas of Infectious Diseases [23], *E. faecium* displays distinctive characteristics and epidemiology compared to *E. faecalis*, along with varying trends across different European countries and regions, showing an upward trajectory over the past two decades. For instance, in the Netherlands, the average number of invasive ampicillin-resistant enterococcal infections per hospital increased fivefold from 1999 to 2005 [24]. By 2007, enterococcal isolates exhibited significant variation, ranging from over 30% in countries such as Greece and Ireland to less than 1% in Scandinavian countries [25]. Meanwhile, a concerning report from Sweden during the period 2007–2009 highlighted an approximately fourfold increase [26].

**Figure 1:**
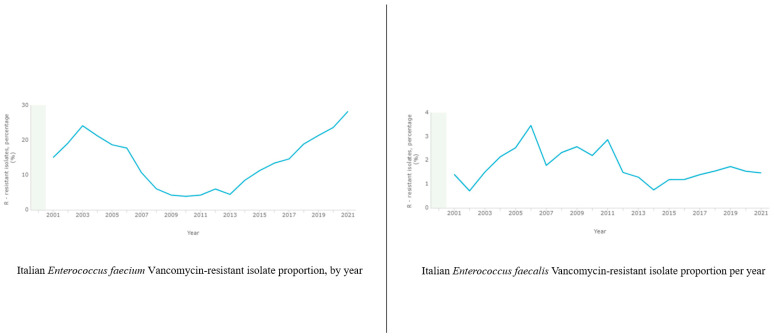
Italian VRE isolate proportion, by year.

**Figure 2:**
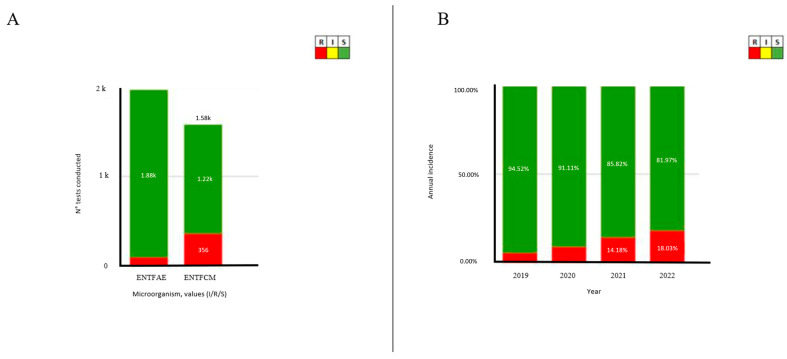
(**A**) Sicilian *Enterococcus* Vancomycin-resistant isolate proportion per species (ENTFAE = *Enterococcus faecalis* and ENTFCM = *Enterococcus faecium* according to RETE MIC Dashboard acronyms). (**B**) Sicilian *Enterococcus faecium* and *faecalis* Vancomycin-resistant isolate proportion per year. Downloaded from https://qlik.qualitasiciliassr.it/anonimo/single/?appid=85ada16c-4b41-4bc6-9ca1-405b8243d0c2&sheet=6ad6f3ac3369-41c5-bd72-792243f9091b&opt=ctxmenu,currsel, accessed on 29 November 2023.

In Italy, *Enterococcus faecium* generally cause healthcare-associated infections. Moreover, it is more commonly related with vancomycin resistance, and exhibits a higher propensity for multidrug resistance than *Enterococcus faecalis* (Figure 1). *Enterococcus* infections pose substantial challenges in clinical management due to their antibiotic resistance and ability to cause severe infections, especially in vulnerable patient populations. Among these populations, we performed a screening in the Southern Italy area using “Sicilian antibiotic resistance dashboard—Rete MIC” [27] (Figure 2). The SARS-Cov-2 pandemia reported a drastic increase in antimicrobial-resistant *enterococci*, probably due to insufficient treatment guidelines and antibiotic overprescription. According to literary data, several regions documented high resistance rates among *Enterococcus* spp., especially related to *E. faecium* [28]. *Enterococcus faecalis* and *Enterococcus faecium* represent the most diffused enterococcal species. However, less common enterococci such as *Enterococcus avium*, *Enterococcus gallinarum*, *Enterococcus durans*, *Enterococcus hirae*, and *Enterococcus raffinosus* can cause more than 24% of enterococcal infections [29].

### 2.1. Nosocomial Transmission

An initial crucial factor leading to nosocomial enterococcal infection appears to be the heightened density of colonization within the gastrointestinal tract [30]. *Enterococci* residing in the gastrointestinal tract can breach the intestinal barrier and traverse the liver, gaining access to the bloodstream. Once in circulation, these pathogens can travel to the heart, potentially leading to infective endocarditis. Environmental contamination from fecal sources, serving as a reservoir for colonization in other patients, along with contamination of the patient’s skin, primarily contributes to urinary tract infections and intravenous catheter-related infections [31]. Comprehending the transmission patterns of *enterococci* within hospital settings is crucial for infection management. The administration of antimicrobials to hospitalized patients frequently establishes VRE dominance in the gastrointestinal tract, thereby promoting the dissemination of these pathogens. Hence normally, intestinal epithelial cells and Paneth cells produce REGIIIγ, a C-type lectin that combats Gram-positive bacteria [32]. REGIIIγ production is initiated by the presence of Gram-negative bacteria and their microorganism-associated molecular patterns (MAMPs), such as lipopolysaccharide and flagellin [33]. However, antibiotic treatment reduces the population of Gram-negative bacteria, resulting in decreased REGIIIγ production. This decline in REGIIIγ secretion creates a favourable environment for *enterococci* to multiply and become the primary constituents of the gut microbiota [32,33]. In addition, the factors contributing to the risk of colonization and subsequent infection with VRE include close physical proximity to infected or colonized patients, prolonged hospitalization, stays in long-term care facilities, surgical or intensive care units, urinary catheterization, and repeated antibiotic treatments [34]. Antibiotic administration often increases the density of VRE in the gastrointestinal tract, facilitating their dissemination through faecal contamination of the hospital environment, including surfaces and the hands of healthcare workers and visitors. Moreover, *enterococci* exhibit remarkable resilience on environmental surfaces such as medical equipment, toilets, bed rails, and doorknobs, and demonstrate tolerance to heat, chlorine, and certain alcohol preparations, enhancing their survival and transmission potential [35].

### 2.2. Molecular Epidemiology in Clinical Setting

Population genetics studies have shed light on the evolutionary dynamics of *enterococci* [36,37]. Analyses employing multilocus sequence typing (MLST), which evaluates allelic variations in seven housekeeping genes, identified distinct clonal complexes among isolates recovered from hospitalized patients, distinguishing them from those of commensal or animal-derived isolates [38]. Notably, hospital-derived *E. faecalis* isolates predominantly cluster in clonal complexes CC2 and CC9 [39], while the increasing prevalence of *E. faecium* is attributed to a polyclonal subpopulation, particularly MLST sequence types 17, 18, 78, and 192, formerly known as clonal complex CC17 [40]. While *E. faecalis* is responsible for the majority of infections, the hospital-adapted genotype of *E. faecium* exhibits a higher propensity for MDR [29]. The global phylogeny of *E. faecium* reveals the prevalence of two discernible phylogenetic clades, labeled A and B. Clade A can be subdivided into two distinct subclades: A1, predominantly composed of clinical strains, and A2, comprising strains primarily isolated from animals but also some non-hospitalized individuals. Clade B encompasses isolates from community settings [41]. The plasticity of enterococcal genomes presents a challenge, with acquired elements potentially accounting for up to a quarter of the genome [42,43]. Conjugation events between *enterococci* can lead to the generation of hybrid strains, resulting in alterations in MLST patterns and genomic composition [44]. Notably, clustered regularly interspaced short palindromic repeats (CRISPR) loci and other genetic markers may contribute to provide insights into the phenotypic traits and genetic makeup of *enterococci*, as well as distinguishing between low-risk and high-risk strains [45].

### 2.3. Clinical Impact

*Enterococci* are one of the most common sources of healthcare-associated infections in developed countries, causing a range of infections such as bacteraemia, infective endocarditis, intra-abdominal and pelvic infections, skin infections, and central nervous infections [46,47]. Intra-abdominal, pelvic, and post-surgery wounds infections are the second most frequent enterococcal infection type. Additionally, *enterococci* rank the third most common causative agent in bloodstream infections and infective endocarditis, leading to significant morbidity and mortality rates [48]. Notably, enterococcal bloodstream infections account for around 10% of all cases of bacteraemia. While extensive studies reveal that *enterococci* are responsible for approximately 30% of hospital-associated endocarditis cases, following *Staphylococcus* spp., *E. faecalis* is the most frequent cause of both bloodstream and urinary tract infections, followed by *E. faecium* [49]. The spread of MDR strains limits treatment options, and *enterococci* have also been isolated from skin infections and, occasionally, reported to cause osteomyelitis, septic arthritis, and central nervous system infections like meningitis. Enterococcal infections, particularly those caused by VRE, are associated with high mortality rates, ranging from 25% to 50%, with a more significant impact on immunocompromised patients [50]. Although initially recognized more as intestinal colonization bacteria than virulent agents, they are increasingly considered causative agents of severe systemic infections, particularly in immunocompromised individuals, including haematological cancer patients. Haematological conditions, such as acute myeloid leukaemia and Hodgkin’s and non-Hodgkin’s lymphoma, that affect the blood and blood-forming organs appear to be related to an increased risk of enterococcal infections. Neutropenic individuals, especially, have a weakened immune response, facing high susceptibility to bacterial infections, including those caused by *Enterococcus* [51,52]. Patients undergoing haematopoietic stem cell transplantation, such as bone marrow or stem cell transplantation, often experience prolonged periods of immunosuppression, carrying vulnerability to various infections, including *Enterococcus* ones [53,54,55,56].

## 3. Antimicrobial Resistance

The use of glycopeptides has been extensively intensified because of the insufficient antimicrobial power of other therapeutical choices. This intensification in glycopeptides administration, along with an avoparcin use increase among farm animals, have boosted resistance diffusion [57]. Since 1995, the European Union (EU) banned the avoparcin use among farms, leading to a diminution in glycopeptide-resistant *enterococci* from human faecal samples. However, data demonstrated how the resistant enterococcal infection prevalence in humans remained elevated [57]. VRE have been a consistent infectious challenge in human medicine since 1980, when the first resistance isolates appeared [58,59]. Furthermore, literary evidence shows the high VRE incidence rates among immunocompromised patients [60]. The glycopeptides resistance is related to the acquisition of *van* genes operons, whose expression allows the production of alternative aminoacidic residuals in the peptidoglycan structure. Specifically, the alternative combinations D-alanin-D-lactate or D-alanin-D-serine substitute the wild-type D-alanin-D-alanin terminus. The substitution preserves the solidity of the enterococcal cell wall, but compromises the target recognition by the glycopeptide drugs. Depending on the involved *van* gene, different resistance phenotypes occur. *VanA* and *VanB* are the most common, followed by less diffused VanD and VanC types. The different Van phenotypes lead to several combinations of antibiotic resistance, alternatively involving vancomycin teicoplanin or both glycopeptide molecules [59,61]. Treating VRE poses a significant challenge due to the limited number of antibiotics available for effective treatment, with linezolid and daptomycin being the primary choices [62]. *Enterococci* are usually broadly susceptible to linezolid, but prior studies have shown that both linezolid- and daptomycin-resistant *enterococci* can emerge after these drug’s exposure or in the absence of their use [63,64,65]. The resistance mechanisms to linezolid are related to rRNA genes, whose number is variable based on the evaluated species (6 for *E. faecium* and 4 for *E. faecalis*). Therefore, the resistance expression level depends on the number of the involved genes. Moreover, *enterococci* can also develop linezolid resistance due to the acquisition of *cfr* genes, which are located in plasmids and contribute to methyltransferase modification [29]. Resistance to daptomycin is often related to phospholipid alterations in enterococcal cell membranes in *E. faecalis* and *E. faecium*. Also, membrane depolarization and cell wall fitness reduction may contribute to the same resistance phenomenon [66].

**Figure 3:**
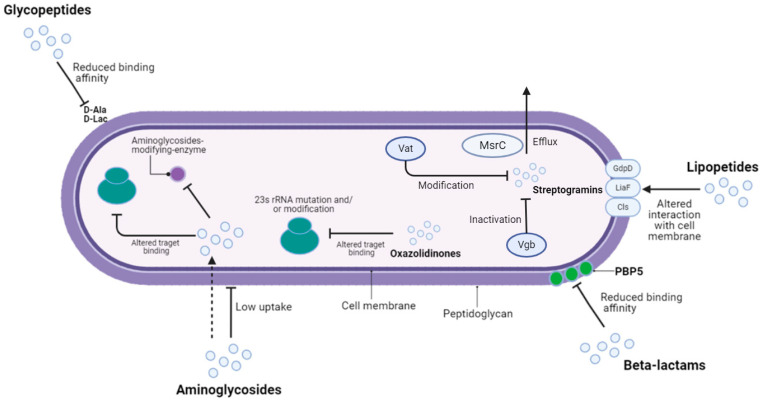
Principal mechanisms of enterococcal antibiotic resistance. The acronyms “*Vat*” and “*Vgb*” indicates streptogramins resistance genes, while “*MsrC*” identifies a macrolides resistance marker. The acronyms “*GdpD*”, “*LiaF*” and “*cls*” indicate regulation system components whose mutations contribute to daptomycin resistance. Finally, the “PBP5” acronym identifies a penicillin-binding protein which express reduced β-lactams affinity. This figure was created by the authors using Biorender.com (app.biorender.com, accessed on 10 December 2023).

*Enterococci* also express resistance against aminoglycosides, β-lactams, and lincosamides [67]. As regards β-lactams resistance, *E. faecalis* often produces β-lactamases, while *E. faecium* expresses high-level penicillin resistance due to the expression of penicillin-binding protein 5 (PBP5). Epidemiological studies suggest how penicillin-resistant *E. faecium* can spread among hospital settings, accounting for more than 70% of incidence. *E. faecalis* strains can be susceptible to carbapenems, which are not a priority therapeutical choice. Some literature evidence highlights the *E. faecalis* capability to develop ceftobiprole resistance through *pbp4* gene sequence alterations, which compromise ceftobiprole binding to its target [68]. The production of aminoglycoside-modifying enzymes and ribosomal mutations leads to intrinsic resistance to aminoglycosides. *Enterococcus* species exhibit mutations in topoisomerase *gyrA* and *parC* genes, leading to an acquired resistance to fluoroquinolones, whose use against enterococcal infections is limited to selected urinary tract infections. While all enterococcal isolates are typically susceptible to erythromycin and tetracyclines, they may occasionally acquire resistance to these antimicrobial agents. For instance, transposons carrying *tet(M)* protection gene mediate tetracycline resistance [29]. *Enterococci* may express macrolides, lincosamides and type b streptogramins (MLSb) resistance due to the acquisition of the *erm* gene, whose expression causes a reduced target affinity for all the MLSb drugs. For this reason, the MLSb phenotype identifies this type of extended antimicrobial resistance [61]. Figure 3 summarizes the principal mechanism of enterococcal antibiotic resistance.

## 4. Virulence Factors

**Figure 4:**
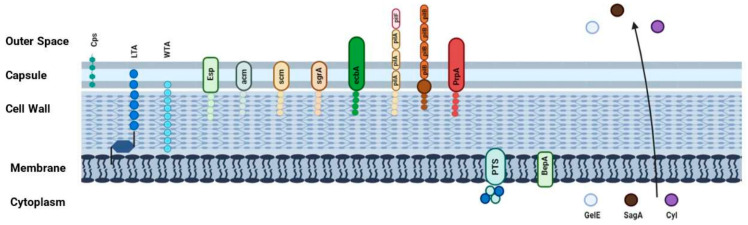
Visual illustration of principal *E. faecalis* and *E. faecium* virulence factors. (this figure was created by the authors using Biorender.com, app.biorender.com, accessed on 10 December 2023).

*Enterococcus* spp. exhibit resilience in harsh environments and diverse ecological niches, transitioning from commensal to pathogen with mechanisms still not fully understood. Intrinsic traits of *E. faecalis*, such as stress responses and antibiotic resistance, likely aid in infection progression [69,70,71]. Bacterial adaptation to environmental changes often involves two-component signal transduction systems (TCS), where a histidine kinase detects signals and transfers a phosphoryl group to a response regulator, regulating gene expression [72]. Among the 17 TCS identified in the genome of *E. faecalis* [73], the Fsr system has been extensively studied and seems crucially connected to enterococcal virulence [74,75,76]. The *fsr* locus encompasses four genes: *fsrA*, *fsrB*, *fsrC*, and *fsrD*, forming a system responsive to extracellular gelatinase biosynthesis-activating pheromone (GBAP) accumulation [77]. FsrB functions as a cysteine protease-like processing enzyme for FsrD peptide [78]. Extracellular FsrD accumulation is sensed by FsrC, activating the response regulator and transcription factor FsrA. Stimulation of FsrC by chemically synthesized GBAP peptide has been demonstrated both in vivo and in vitro [79]. FsrABDC proteins play a pivotal role in activating two *E. faecalis* virulence-associated proteases, gelatinase (GelE) and serine protease (SprE), at a promoter upstream of the *gelE* gene [78,80]. Several studies on animal models have demonstrated the crucial role of these key factors in enterococcal virulence [81,82,83]. Additionally, gelatinase is essential for facilitating efficient biofilm formation and plays a significant role in the pathogenesis of enterococcal endocarditis [84,85]. The FsrA response regulator, similar to AgrA of *Staphylococcus aureus*, belongs to a protein family characterized by a LytTR DNA-binding domain, which is unique compared to the typical DNA-binding domains of response regulators and is frequently associated with the regulation of virulence factors such as toxins, bacteriocins, and extracellular polysaccharides [86]. These virulence factors can facilitate tissue invasion, promote immune evasion, and contribute to the overall virulence of the bacterium [87,88]. In *E. faecium*, extensively studied virulence genes involve surface proteins (Fms) crucial for adhesion, biofilm formation, and pili assembly. Specifically, these proteins include Esp (enterococcal surface protein), Acm (adhesin of collagen from *Efm*), Scm (second collagen adhesin from *Efm*), SgrA (serine-glutamate-repeat-containing-protein A), and EcbA (Efm-collagen-binding-protein A) [89,90,91]. Enterococcal cell membrane glycolipids and lipoteichoic acid play a significant role in pathogenesis [92]. A mutant *E. faecalis* strain lacking the glycolipid α-diglycosyl diacylglycerol (DGlcDAG) showed reduced adherence to enterocytes and biofilm formation [93]. Another virulence factor, SagA, identified as a major antigen in *E. faecium*, comprises three domains with distinct sequence and/or structural characteristics. SagA plays a crucial role in bacterial growth, potentially influencing cell wall metabolism, and can form oligomers when overexpressed, while also binding to fibrinogen and various ECM proteins, indicating broad adhesion capabilities and emphasizing its significance in bacterial virulence. [94]. The *SagA* gene, situated in a gene cluster involved in cell wall metabolism alongside MreCD proteins, encodes a protein sharing sequence homology with cell wall metabolism-related proteins from other bacteria, suggesting its potential involvement in cell wall metabolism. However, its hydrolase activity and structural characteristics differ from those of related proteins [94,95]. Some strains of *E. faecalis* produce a post-translationally modified antimicrobial peptide called cytolysin, capable of lysing both bacterial and eukaryotic cells, thereby contributing to pathogenesis [96]. Xiong et al. discovered two previously uncharacterized small β-barrel Pore Forming Toxins (PFTs), resembling members of the haemolysin family found in human samples, and identified them as part of a broader family of β-barrel PFTs in *E. faecalis*, *E. faecium*, and *E. hirae*. The most potent among these toxins exhibit binding affinity to HLA-I in humans and MHC-I in other animals, establishing them as a significant family of canonical protein toxins in *enterococci* [97]. *Enterococcus* virulence factors, prevalent in clinical isolates, include the carbohydrate phosphotransferase system (PTS), enabling glucose and alternative carbon nutrient uptake [98]. Regulatory proteins termed PTS-regulatory-domain-containing virulence regulators (PCVRs), phosphorylated via the PTS, are vital for bacterial adaptation to hostile and nutrient-limiting host environments [99]. Studies on PCVRs, such as MafR from *E. faecalis*, reveal that loss of MafR significantly diminishes virulence in a murine peritonitis model, suggesting a link between MafR and *E. faecalis* virulence [100]. Additionally, the biofilm and endocarditis-associated permease A (*BepA*) gene, part of PTS and highly expressed in *E. faecium* hospital-associated isolates [101] has been associated with endocarditis in a rat model. It has been demonstrated that BepA contributes to in vitro biofilm formation of *E. faecium* in the presence of human serum and to the metabolism of β-methyl-D-glucoside [102]. Figure 4 illustrates the main *E. faecium* and *E. faecalis* virulence factors. Additionally, Table 1 summarizes their biological characteristics and actions.

## 5. Interactions in Immunocompromised Host

The immune system is a complex network of molecules, cells, tissues, and organs that plays a crucial role in recognizing and eliminating foreign agents while maintaining a stable internal environment [121,122,123]. In individuals with intact immune systems, the detection of *Enterococcus* typically activates the host’s immune response, leading to the recruitment of immune cells like neutrophils and macrophages at the infection site for bacterial elimination, accompanied by the release of cytokines, chemokines, and antimicrobial peptides to combat the infection [121]. However, individuals with compromised immune systems, such as those undergoing immunosuppressive therapy or with certain underlying conditions, are more susceptible to *Enterococcus faecium* and *Enterococcus faecalis* infections [124,125,126,127]. Immunocompromised individuals are at an elevated risk of developing drug-resistant enterococcal infections, primarily because they frequently receive broad-spectrum antibiotics in hospital settings where resistant strains are prevalent [31,128]. Patients undergoing treatments like chemotherapy and hematopoietic stem cell transplantation face a heightened susceptibility due to their compromised immune systems, which include reduced innate immunity and weakened mucosal barriers [129]. These factors make it easier for multidrug-resistant organisms like VRE to colonize the skin and intestinal tract, eventually leading to infections [130]. The use of central venous catheters in hospitals provides an additional pathway for nosocomial pathogens to enter the bloodstream during hospitalization. When bacteria enter the bloodstream, they encounter different conditions compared to those in the skin or intestines. These conditions include altered nutrient availability, varying host immune responses, and fluctuating antibiotic concentrations [131]. Such factors create selective pressures that favour the growth of bacterial variants better suited for survival and proliferation in the bloodstream [132]. Previous studies involving immunocompromised patients revealed that VRE adapted to the bloodstream environment by activating a bacterial stringent response. This adaptation rendered antibiotics less effective in treating the infection, and resolution only occurred after immune function was restored through a granulocyte infusion [13,120].

### 5.1. Clinical Risk-Factors and Predictors of Mortality in Host

The correlation between VRE colonization or infection and specific pre-existing medical conditions such as diabetes mellitus, chronic renal failure, cancer, and transplantation is widely recognized [133,134,135,136,137,138,139]. Hospitalization in wards predominantly treating patients with hematological diseases is also linked to VRE colonization. This connection is compounded by the extensive use of broad-spectrum antibiotics in these wards, resulting in heightened colonization pressure and increased VRE transmission [133,140]. The presence of an invasive device has previously been identified as a significant clinical risk factor for VRE invasive infections [134]. Invasive procedures such as catheter insertion, ventilation, and surgery increase the risk by providing entry routes for *Enterococcus*, which can form biofilms on medical devices, or merely serve as indicators of debilitation, prolonged hospital stays, and severe comorbidities [141,142,143,144]. Patients with hematological conditions and invasive devices, undergoing prolonged antibiotic therapy in the same ward, may have acted as VRE reservoirs. Previous studies emphasize the significance of close proximity to culture-positive patients for VRE spread, such as Byers et al., that highlighted the proximity to non-isolated, colonized patients as a key risk factor for VRE acquisition [145]. Moreover, they suggested that while antibiotic exposure is crucial, it may not be adequate alone to yield a positive VRE culture in patients without prior VRE exposure.

Numerous studies have explored whether prior antimicrobial therapy serves as a risk factor for nosocomial VRE, with divergent results [146]. The main agents reported include vancomycin, cephalosporins, and antimicrobials targeting anaerobic organisms [141,147,148,149,150,151,152]. Continuous vancomycin administration has been proposed to create an intestinal niche conducive to VRE proliferation, potentially heightening the risk of VRE bloodstream invasion [149]. Glycopeptide usage might facilitate VRE carriers in transitioning to transmitters rather than heightening the likelihood of non-carriers becoming colonized [146]. Sakka et al. outline link between exposure to antimicrobial agents with anti-anaerobic properties and VRE colonization [140]. These medications potentially enhance VRE colonization in the lower gastrointestinal tract by suppressing anaerobic flora in the gut [136,147]. Consequently, individuals exposed to such agents are at an increased risk of VRE bloodstream infections. Therefore, it is suggested that restricting the use of antimicrobial agents with anti-anaerobic activity could aid in reducing the transmission and spread of VRE [21]. Hospitalization in a medical ward or admission to the ICU within the past 6 months are additional risk factors associated with VRE colonization in high-risk environments [134,153]. Research indicates that VRE colonization is often found in patients who later develop bacteremia. Moreover, in studies with immunocompromised patients, VRE colonization demonstrated a high negative predictive value (99.9%) and a positive predictive value of 29.3% for predicting the development of bacteremia [135]. VRE bacteremia can arise from prolonged gastrointestinal colonization, often occurring as an isolated breakthrough event amidst intense antimicrobial therapy. Invasive VRE infections are linked to significant morbidity and mortality rates [12,154]. Studies have previously linked the elevated crude mortality rate seen in VRE-infected patients to the severity of underlying illness [155].

### 5.2. Enterococcus Pathogenicity in Host Cells

The discovery of a widely distributed clonal lineage of the virulent *E. faecium* strain (CC-17), adapted to hospital environments, has revealed the swift dissemination of resistant strains within healthcare settings. The acquisition of ampicillin resistance and the putative *esp* pathogenicity island by *E. faecium* have bolstered its capacity to thrive in hospitals, enabling transmission and contributing to nosocomial outbreaks [40]. Comparative and functional genomics may detect indicators of VRE adaptation during colonization of the gastrointestinal tract and bloodstream infection in immunocompromised individuals [156]. Recent research on VRE adaptation to the human intestinal tract has also detected changes in carbohydrate metabolism during extended colonization periods [157]. Chilambi et al. report that a usual adjustment in carbohydrate utilization was related to the independent occurrence of a Y585C mutation in the sorbitol operon transcriptional regulator *gutR* across various STs [120]. The *gut* operon is more prevalent among hospital-adapted *E. faecium* isolates than commensal ones [156]. Therefore, sorbitol exposure, due to its use as a dietary sugar substitute and laxative in chemotherapy patients, might have contributed to the emergence and prevalence of the gutR Y585C mutation in VRE strains, a phenomenon observed in immunocompromised settings.

Typically, researchers identify common trends in how VRE adapts across various genetic backgrounds and establish connections between these genetic alterations and clinically differences in traits [40,62]. In particular, the discovery of significant genetic rearrangements, such as chromosomal inversions exceeding 1 Mb in size and similar genomic alterations, have been observed previously in the genomes of *E. faecium* associated with hospital environments [158,159]. However, the exact role and mechanisms through which these rearrangements may impact the bacterium’s ability to survive in a hospital setting still require further investigation.

Several studies demonstrated the ability of *Enterococcus* to influence multiple signaling pathways within host cells. Boonanantanasarn et al. found that *E. faecalis* induces EGFR activation in oral cancer cells through hydrogen peroxide (H_2_O_2_) production, either directly by the bacteria or via metalloproteinase-dependent EGF-like signals [160]. EGFR activation can significantly contribute to MEK/ERK activation, promoting cell proliferation [161,162,163]. The ability of gefitinib and catalase to attenuate *E. faecalis*-induced EGFR activation and cell proliferation, along with the inhibition of TGF-α activation by these inhibitors, suggests potential clinical applications for catalase and EGFR inhibitors in treating *E. faecalis*-induced oral carcinogenesis [160]. In experiments with HCT-116, an aggressive colorectal cancer cell line, *E. faecalis* was found to downregulate the expression of the *FIAF* gene (angiopoietin-like protein 4), which is typically associated with the development of certain cancer types [164,165]. In a mouse model of ulcerative colitis, an increase in *E. faecalis* colonization following vinegar treatment was associated with inflammation inhibition by suppressing T helper (Th)-1 and Th17 responses [166]. Additionally, in studies using human peripheral blood mononuclear cells, the heat-killed *E. faecalis* YM-73 strain exhibited immunomodulatory effects, increasing Th1-associated cytokines while reducing Th2-associated cytokines [167]. Wang et al. showed that *E. faecalis* Lipoteichoic Acid (LTA) induces the expression of the pro-inflammatory cytokine TNF-α by activating the p38 MAPK and NF-κB signaling pathways in differentiated THP-1 macrophages [168]. In addition, some studies have explored the potential involvement of miRNAs in *Enterococcus* infections. For instance, Li et al. investigated the role of miR-200a-3p in BMSC migration stimulated by E. faecalis supernatants, along with its downstream target FOXJ1. They also identified activation of the NFκB pathway, which promoted migration by upregulating MMP-3 and MMP-13 expressions. These findings offer a new perspective on the potential role of miRNAs in *Enterococcus*-host cell interactions [169]. Nevertheless, further research is needed to uncover the specific mechanisms and implications of miRNA regulation in *Enterococcus* infections, as well as to elucidate the broader role of miRNAs in the interplay between bacteria and host cells. In conclusion, all these pathways play critical roles in diverse cellular processes such as cell proliferation, survival, and inflammation. However, it’s likely that *enterococci* exert influence over additional signalling pathways that have yet to be elucidated, highlighting the complexity of their pathogenicity.

### 5.3. Biofilm Formation and Adhesion to Immunocompromised Host Tissues

*Enterococcus faecalis* is well-known for its ability to form biofilms [170,171]. Biofilms are structured communities of bacteria enclosed in a self-produced matrix. Biofilm formation involves the production of extracellular polymeric substances (EPS), which provide structural support and protect bacteria from host immune defenses and antimicrobial agents [172]. Several studies examined alterations in genes known to play a role in biofilm formation in *E. faecium*, such as *ebpABC* (biofilm-associated pilus), *esp* (enterococcal surface protein), *asrR* (antibiotic stress and response regulator), *acm* (collagen adhesion protein), and *atlA* (autolysin) [173]. Within biofilms, *Enterococcus faecalis* can attach to surfaces, such as medical devices or damaged tissues, shielding host immune responses and antibiotics. This capability makes biofilm-related infections challenging to eradicate in several clinical settings [174,175]. An interesting study by Fiorotto et al. highlighted how *Enterococcus* can interact with components of the host’s extracellular matrix, such as collagen and fibronectin, in an organoid model [176]. Therefore the interactions promote bacterial adhesion to host tissues and may contribute to the formation of biofilms [87,177,178]. The understanding of capsular polysaccharide (*cps*) in *E. faecium* is presently limited, with minimal knowledge extending beyond a potential capsule biosynthetic locus, which exhibits variability among different sequence types [37]. The literature reported that mutations in the *cps* locus may result in production changes of cell surface-associated polysaccharides. These changes could lead to increased biofilm formation, enhancing the colonization or persistence of VRE isolates carrying these mutations. Biofilm formation, in turn, amplifies bacterial infectivity and disease-causing potential by enhancing adhesion to host tissues and medical devices like catheters, increasing resistance to phagocytosis, and bolstering resilience against antibiotics [179,180]. Furthermore, the composition of enterococcal cell surface-associated polysaccharides has been demonstrated to influence bacterial sensitivity to lysozyme [181]. Lysozyme is a crucial component of the innate immune response. Lysozyme acts as a hydrolase, degrading the glycosidic bonds between N-acetylmuramic acid (NAM) and N-acetylglucosamide (NAG) in peptidoglycan in the bacterial cell wall, resulting in increased cell wall permeability and ultimately cell death [182]. The deletion of *RasP* (*CdRasP*) and *RseP* (*EfsRseP*), the site-2-metalloproteases of respectively *E. faecalis* and C. difficile, results in a decreased activity of σV, which is the extracytoplasmic function sigma factor which mediates the lysozyme resistance. This activity reduction consequently leads to a decreased lysozyme resistance [183,184,185]. Similar observations have been made in *Enterococcus faecium*, where mutations within *RseP* results in a 6–8-fold increase in lysozyme susceptibility as reductions in desiccation tolerance [183,186]. Therefore, the ability to tolerate constitutive innate immune system defenses such as lysozyme could have also played a role in selecting about capsule-lacking, biofilm-forming bacteria [183,187].

## 6. Therapeutical Approaches

### 6.1. Treatment for Susceptible Enterococci

Susceptible enterococcal strains commonly respond to β-lactam and aminoglycoside combinations. In order to reduce aminoglycoside side effects such as nephrotoxicity and ototoxicity, clinical guidelines also propose a double β-lactams therapy [188]. The synergic effect between amoxicillin and cefotaxime has been extensively demonstrated, along with the minimum inhibitory concentration (MIC) decrease in the case of simultaneous amoxicillin and ceftriaxone usage. Although *Enterococcus* intrinsic cephalosporins resistance, the association with ampicillin leads to penicillin-binding proteins (PBPs) inactivation, justifying the success of the combination. Specifically, several endocarditis episodes showed a reduced vegetation after this therapeutic regimen [189].

### 6.2. Challenges in Treatment of VRE Infection

VRE present challenges in treatment, particularly as resistance often emerges in strains of *E. faecium* already MDR [190]. These strains typically exhibit resistance to multiple antimicrobial drugs, including erythromycin, tetracycline, fluoroquinolones, and rifampicin, and do not respond synergistically to aminoglycosides [191]. Determining optimal therapy for infections caused by such strains remains uncertain. Physicians may resort to administering drugs or combinations with proven in vitro activity. However, discerning whether specific therapy is necessary for enterococcal isolates or assessing the infection’s role in the patient’s outcome relative to underlying disease can be challenging. Evaluating antimicrobial therapy efficacy for VRE infection is complicated by underlying disease severity and concurrent interventions. Nevertheless, literature suggests several antimicrobial drugs may offer benefits to patients infected with VRE, based on in vitro studies, animal research, anecdotal case reports, and small, uncontrolled series over the past decades [192,193,194,195,196]. The resistance mechanisms require new therapeutical strategies, which could be limited and related to possible complications [197,198]. VRE infections result in increased morbidity, mortality, and medical costs, necessitating a comprehensive treatment strategy involving all infection control professionals [199]. Certainly, the advent of VRE strains highlights the pressing need for brand-new antimicrobial drugs [200,201]. Treatment options for VRE infections mainly include linezolid as the only FDA-approved therapeutical choice. Off-label therapeutical strategies may involve daptomycin, often combined with fosfomycin or β-lactams, oritavancin, tigecycline, and tedizolid. All these agents are not approved yet in the case of VRE isolations due to the lack of extended clinical studies about their effectiveness. According to literature data, high daptomycin dosage may be bactericidal against VRE. However, some *E. faecium* isolates tend to survive those regimens, requiring a daptomycin combination with β-lactam antibiotics [202]. The daptomycin bactericidal activity is enhanced by the addition of carbapenems such as ertapenem or cephalosporins such as cefepime, ceftriaxone, and cefazolin. Moreover, the association of ceftaroline and daptomycin demonstrated high success percentage in *E. faecium* and *E. faecalis* infection treatment [203]. Previous studies have considered the role of tigecycline in VRE infection treatment, documenting an activity in the case of soft tissue and intra-abdominal infection episodes. However, further clinical trials will be essential to optimize the tigecycline regimen for those complicated *Enterococcus* spp. Infections [204].

Quinupristin/dalfopristin may represent a valid alternative against *E. faecium* due to *E. faecalis* intrinsic resistance to this double streptogramins combination [205]. Regarding new cephalosporins, ceftaroline and ceftobiprole demonstrated significant in vitro against the most common agents of infective endocarditis, including *Enterococcus faecalis* [206].

### 6.3. Prevention and Infection Control Strategies

Enterococcal antimicrobial resistance and virulence underscore the importance of controlling these microorganisms’ diffusion, especially among the hospital recovered patients. Reccomandation for controlling VRE involve isolating individuals with current or past VRE colonization, using protective gown and gloves, hand hygiene with antiseptic post glove removal, and allocating dedicated equipment like stethoscopes for VRE-colonized patient rooms [207]. Additionally, precautions should be taken to avoid touching environmental surfaces (e.g., doorknobs) after glove removal. Enhanced environmental cleaning methods may be recommended due to documented instances of persistent VRE presence in hospital rooms despite standard cleaning procedures [207,208]. Recent studies have demonstrated that decolonization through the use of chlorhexidine gluconate bathing for patients in Intensive Care Units (ICU) can prevent hospital-acquired infections and reduce the acquisition rate of MDR organisms including VRE [209]; this could be a potentially interesting path to follow for wards characterized by the presence of immunocompromised patients. Published data highlight the possibility of using capsular and cell wall polysaccharides as targets for immunotherapeutic choices. Specifically, these carbohydrates could be used as targets for antibodies, contributing to *E. faecalis* and *E. faecium* killing. Furthermore, the isolation of polysaccharides from enterococcal-grown colonies has been experienced in vaccine development and production [210]. The European Antimicrobial Resistance Surveillance Network (EARS-Net) currently include *E. faecalis* and *E. faecium* within its alert pathogens list. This inclusion in the network encourages the microbiology laboratories in furnishing identification and precise susceptibility profiles [211]. VRE poses a significant threat in healthcare settings, warranting the implementation of antimicrobial stewardship programs. Enhanced surveillance and screening have been effective in identifying colonized individuals and implementing necessary preventive measures [212,213,214]. Several studies underscore the importance of active rectal swab screening for detecting VRE strains. In ICU settings, surveillance swabs aid in patient monitoring, reducing VRE transmission to sterile sites. A positive VRE result might forecast glycopeptide resistance in subsequent infections [215,216,217]. We advocate for stringent surveillance, particularly in wards with immunocompromised patients.

## 7. Conclusions

*Enterococci* are significant human pathogens with diverse characteristics and clinical implications. Understanding the intricate interactions between *Enterococcus* and immunocompromised individuals is crucial for effective infection management. These patients, highly susceptible to *Enterococcus* infections, including drug-resistant strains like VRE, require enhanced surveillance and strict infection control measures, particularly in high-risk settings such as haematology, ICUs, and other wards with immunocompromised patients. Identifying clinical risk factors and predictors of mortality associated with VRE is vital for early intervention and optimal patient outcomes. Although therapeutic approaches for VRE infections present challenges, ongoing research aims to develop new strategies and treatments. Prevention and control strategies, including antimicrobial stewardship programs and environmental cleaning, are essential to curb VRE spread and reduce infection burden in healthcare settings. Despite continuous research efforts, it is necessary to address the complex challenges posed by *Enterococcus* infections. Our hope is to utilize these findings to develop more efficient approaches for managing and containing enterococcal infections in susceptible patient populations.

## Figures and Tables

**Table 1:** Summary of *E. faecalis* and *E. faecium* virulence factors in terms of biological characteristics and actions.

Virulence Factor	Description	References
Secreted factors		
Cyl, haemolysin–cytolysin	Shows broad-spectrum binding to the extracellular matrix and is crucial for cell growth Contributes to biofilm formation and lysis of red blood cells, macrophages and polymorphonuclear neutrophils	[103,104,105,106]
SagA, secreted antigen A	Shows broad-spectrum binding to the extracellular matrix and is crucial for cell growth Contributes to biofilm formation Highly immunogenic and a promising vaccine candidate	[94,107]
GelE, gelatinase	Plays a role in virulence in animal models of endocarditis, peritonitis, and endophthalmitis Affects adherence, autolysin, and biofilm formation Regulated by the Gsr quorum sensing system Highly prevalent in the predominant CC17 clinical clones	[75,77,80,81,85]
sprE, serine protease	Serine protease that allow the pathogen to spread to cells and degrade connective components of tissue matrices. These invasin can also be thought of as spreading factors that allow the bacteria to move throughout the host.	[108,109]
Cell surface determinants and their formation		
Agg, aggregation substance	Aggregation protein involved in adherence to eukaryotic cells Cell aggregation and conjugation Transfer and survival of some plasmids in neutrophils Role in *E. faecalis* endocarditis	[110,111,112,113]
efaA, *Enterococcus faecium*/*faecalis* antigen A	Cell wall adhesins expressed in serum by *E. faecalis* and *E. faecium*	[114,115]
Esp, Enterococcal surface protein	Contributes to forming biofilms and attaching to silicon-based surfaces May be associated with *cyl* genes on a pathogenicity island Acts as a virulence factor in UTI and endocarditis in animal models Cell wall-associated protein involved in immune evasion	[89,90,91]
Ace and Acm, collagen-binding proteins	Contributes to the formation of biofilms Acts as a virulence factor in UTI and endocarditis in animal models Mediate adherence to collagen (Acm), and to collagen and laminin (Ace)	[89,90,91,116]
Scm, second collagen adhesin from *E. fm*	Binds to various ligands of the extracellular matrix Highly prevalent in clinically related *E. faecium*	[89,90,91]
SgrA, EcbA	Surface-binding adhesins Implicated in biofilm formation Both serve as a markers for clinically associated *E. faecium*	[89,90,91,117]
epa, enterococcal polysaccharide antigen	Affect biofilm formation and translocation across intestinal epithelial cells Role in experimental peritonitis and UTIs Affect bacterial cell susceptibility to killing by polymorphonuclear neutrophils	[118]
Ebp, endocarditis- and biofilm-associated pili	Form pili Mediate adherence to platelets, fibrinogen and collagen Linked to infection Contribute to adherence in biofilm formation and during UTIs	[89,114,115,119]
Other factors		
Cps, capsular polysaccharides	Involved in adherence, virulence, and evasion of phagocytosis. Crucial components for immune response	[120]
LTA, lipoteichoic acid	Component of the cell wall Attached to lipid membrane Contributes to resistance against microbes, biofilm formation, and virulence.	[92,93]
WTA, wall teichoic acid	Resists neutrophil killing mediated by complement Involved in attaching to host cells Contributes to beta-lactam resistance	[92,93]
Fsr regulator locus	The main regulatory system for quorum sensing in *E. faecalis*, it consists of the genes *fsrA*, *fsrB*, *fsrD*, and *fsrC*, which control the expression of gelatinase and serine protease. Through this Fsr quorum-sensing system, biofilm formation is governed by the regulation of gelatinase production.	[74,75,76,77]
PTS, carbohydrate phosphotransferase system	Predominantly found in Clade A clinical isolates Plays a role in intestinal colonization in a mouse model BepA (PTS) contributes to in vivo biofilm formation and is associated with endocarditis in a mouse model	[99,100,101,102]
Ddl, D-Ala-D-Ala ligase	Non-vancomycin ligases that produce D-Ala-D-Ala, the standard cell wall precursor that increases susceptibility to glycopeptide antibiotics. Changes in the *ddl* gene may lead to the production of ineffective or deactivated D-Ala-D-Ala ligases, potentially making bacteria reliant on glycopeptides based on the existence of vancomycin resistance clusters.	[45,87]
